# Chemistry and Metallurgy Applied to Dentistry

**Published:** 1899-10

**Authors:** 


CHEMISTRY AND METALLURGY APPLIED TO DENTISTRY. By Vernon J. Hall, Ph.D., Professor of Chemistry and Director of Chemical Laboratories in the Dental School and in the Woman's Medical School of Northwestern University. Published by the Technical Press at Evanston, Illinois, 1898.

The subject of Dental Metallurgy is, comparatively speaking, a new one. From the very earliest date in the history of dentistry metals have been used to fulfil the various indications. This use of the metals, however, has been, and still is, to a great extent an empirical one. Recently one or two of the leading dental colleges have striven to rid the science of dental metallurgy of some of those mysteries hovering around it, by establishing practical courses in which the physical and chemical properties of the metals are taught, and where they can actually be observed.

This practical study, having so recent a birth, requires, like all other things, development. The literature on the subject must necessarily be sparse, therefore any new book on this topic must be received with gratification, as it may give new points that will further the study of this branch of dental work.

This book by Dr. Hall can be read with great profit by the student, as it contains many valuable points. Dr. Hall is very explicit in some things, but not enough so in others. A short description of the phenomena attending the "glancing" of a gold or silver button is rather an essential, otherwise how is a student to know when the last trace of lead has disappeared. When writing that the cupel should be heated a few moments before using, it should be added that the cupel must be made of pure bone-ash, for the ordinary commercial cupel certainly requires to be thoroughly heated for some time, in order to be on the safe side.

It is hardly necessary to weigh the test lead,—measuring is sufficiently accurate. Neither is it fair to use the term assay ton without some explanation of what it is, and what object led Dr Chandler to suggest this system of weights.

